# Assessment of an Instrument to Measure Interdisciplinary Staff Perceptions of Quality of Dying and Death in a Pediatric Cardiac Intensive Care Unit

**DOI:** 10.1001/jamanetworkopen.2022.10762

**Published:** 2022-05-06

**Authors:** Valerie Bailey, Dorothy M. Beke, Jennifer M. Snaman, Faraz Alizadeh, Sarah Goldberg, Melissa Smith-Parrish, Kimberlee Gauvreau, Elizabeth D. Blume, Katie M. Moynihan

**Affiliations:** 1 Cardiovascular and Critical Care Nursing Patient Services, Boston Children’s Hospital, Boston, Massachusetts; 2 Department of Psychosocial Oncology and Palliative Care, Dana-Farber Cancer Institute, Boston, Massachusetts; 3 Department of Pediatrics, Boston Children’s Hospital, Boston, Massachusetts; 4 Department of Cardiology, Boston Children’s Hospital, Boston, Massachusetts; 5 Department of Pediatrics, Harvard Medical School, Boston, Massachusetts; 6 Department of Critical Care Medicine, Vanderbilt, Nashville, Tennessee; 7 Sydney Medical School, University of Sydney, Sydney, Australia

## Abstract

**Importance:**

Lack of pediatric end-of-life care quality indicators and challenges ascertaining family perspectives make staff perceptions valuable. Cardiac intensive care unit (CICU) interdisciplinary staff play an integral role supporting children and families at end of life.

**Objectives:**

To evaluate the Pediatric Intensive Care Unit Quality of Dying and Death (PICU-QODD) instrument and examine differences between disciplines and end-of-life circumstances.

**Design, Setting, and Participants:**

This cross-sectional survey included staff at a single center involved in pediatric CICU deaths from July 1, 2019, to June 30, 2021.

**Exposures:**

Staff demographic characteristics, intensity of end-of-life care (mechanical support, open chest, or cardiopulmonary resuscitation [CPR]), mode of death (discontinuation of life-sustaining therapy, treatment limitation, comfort care, CPR, and brain death), and palliative care involvement.

**Main Outcomes and Measures:**

PICU-QODD instrument standardized score (maximum, 100, with higher scores indicating higher quality); global rating of quality of the moment of death and 7 days prior (Likert 11-point scale, with 0 indicating terrible and 10, ideal) and mode-of-death alignment with family wishes.

**Results:**

Of 60 patient deaths (31 [52%] female; median [IQR] age, 4.9 months [10 days to 7.5 years]), 33 (55%) received intense care. Of 713 surveys (72% response rate), 246 (35%) were from nurses, 208 (29%) from medical practitioners, and 259 (36%) from allied health professionals. Clinical experience varied (298 [42%] ≤5 years). Median (IQR) PICU-QODD score was 93 (84-97); and quality of the moment of death and 7 days prior scores were 9 (7-10) and 5 (2-7), respectively. Cronbach α ranged from 0.87 (medical staff) to 0.92 (allied health), and PICU-QODD scores significantly correlated with global rating and alignment questions. Mean (SD) PICU-QODD scores were more than 3 points lower for nursing and allied health compared with medical practitioners (nursing staff: 88.3 [10.6]; allied health: 88.9 [9.6]; medical practitioner: 91.9 [7.8]; *P* < .001) and for less experienced staff (eg, <2 y: 87.7 [8.9]; >15 y: 91, *P* = .002). Mean PICU-QODD scores were lower for patients with comorbidities, surgical admissions, death following treatment limitation, or death misaligned with family wishes. No difference was observed with palliative care involvement. High-intensity care, compared with low-intensity care, was associated with lower median (IQR) rating of the quality of the 7 days prior to death (4 [2-6] vs 6 [4-8]; *P* = .001) and of the moment of death (8 [4-10] vs 9 [8-10]; *P* =.001).

**Conclusions and Relevance:**

In this cross-sectional survey study of CICU staff, the PICU-QODD showed promise as a reliable and valid clinician measure of quality of dying and death in the CICU. Overall QODD was positively perceived, with lower rated quality of 7 days prior to death and variation by staff and patient characteristics. Our data could guide strategies to meaningfully improve CICU staff well-being and end-of-life experiences for patients and families.

## Key Points

**Question:**

In the absence quality metrics for end-of-life care in pediatric cardiac intensive care units (CICUs), how do interdisciplinary staff perceive quality of dying and death (QODD)?

**Findings:**

In this cross-sectional survey study of 713 medical professionals involved in 60 deaths in the CICU, the pediatric intensive care unit (PICU)–QODD instrument was a reliable and valid measure of QODD in CICUs, with overall positive perceptions of QODD yet negative perceptions of the 7 days prior. Lower PICU-QODD scores were reported by nursing or allied health staff, by less experienced staff, for patients with cardiac-surgical admissions and comorbidities, and for deaths following treatment limitation or misaligned with family wishes.

**Meaning:**

These data could guide strategies to improve staff well-being and end-of-life experiences.

## Teaser

This cross-sectional study evaluates the Pediatric Intensive Care Unit Quality of Dying and Death instrument and examines differences between disciplines and end-of-life circumstances.

## Introduction

Despite medical and surgical advancements, congenital and acquired heart disease remains a leading cause of childhood mortality.^1^ A high proportion of pediatric cardiac deaths occur in cardiac intensive care units (CICU), and end-of-life care is a driver of moral distress and burnout among health care professionals, emphasizing imperative of research in this setting.^2,3,4,5,6,7,8,9,10^ Providing end-of-life care for children and their families in the complex CICU environment is particularly challenging.^2,3,4,5,11,12,13,14,15^ First, the increased use of technology has amplified prognostic uncertainty, with treatment often at the nexus between comfort and burdensome therapies. Second, interdisciplinary (ie, cardiology, cardiothoracic surgery, intensive care) and interprofessional (nursing, allied health, medical) practitioners with differing experience, perceptions of benefits and burdens, and acceptability of outcomes work together. Third, responsibility for decision-making for pediatric CICU patients is shared by families and teams.^16^ Furthermore, low mortality means survival is expected, even in severe critical illness.^17^ Finally, childhood death violates the natural generational cycle and results in profound grief in bereaved families. These complexities contribute to conflict, may adversely affect staff perceptions of end-of-life care, and contribute to moral injury.

Quality of dying and death (QODD) is a construct that evaluates perceptions of end-of-life care with measurement tools validated for adults and pediatric intensive care units (PICUs).^18,19^ CICU staff play an integral role supporting children and their families at end of life, yet despite complexity, no studies, to our knowledge, have examined interdisciplinary perceptions of CICU QODD, and no universally accepted end-of-life outcome measures exist in this setting.^2,5,13,15,20^ In the absence of quality indicators and with challenges ascertaining family perspectives, staff perceptions of the quality of end-of-life care are highly valuable to inform optimal care delivery.^13,21^

Exploring interdisciplinary perceptions of QODD offers an opportunity to identify strategies to positively affect end-of-life experiences for children with heart disease and their families and staff well-being.^22^ We evaluated staff perceptions of QODD in the CICU and differences between disciplines and end-of-life circumstances.

## Methods

We performed a single-site cross-sectional survey and medical record review at a quaternary pediatric CICU with ethics approval from the Boston Children's Hospital Institutional Review Board, Clinical Investigation, and consent implied from voluntary survey completion. This report follows the Strengthening the Reporting of Observational Studies in Epidemiology (STROBE) reporting guideline for cross-sectional studies. All CICU deaths over a 2-year period (July 1, 2019, to June 30, 2021) were included. Interdisciplinary staff directly involved in patient management in the 72 hours before death or as continuity clinicians (attendings, fellows, or nurse practitioners [NPs]) were identified by the bereavement coordinator, approached, and invited to participate. Staff included bedside nurses, allied health staff (including social workers, child-life specialists, respiratory therapists, and extracorporeal membrane oxygenation [ECMO] specialists), and medical practitioners (including CICU attending physicians, cardiology and surgery attendings, CICU NPs, and fellows) (Table 1). Responses were collected within 14 days of the child’s death. Staff self-reported limited demographic data to ensure anonymity. Survey responses had a unique patient-level study identifier and were deidentified following linkage.

**Table 1.** Patient Characteristics, End-of-Life Circumstances, and Staff Demographic Characteristics

Characteristic	No. (%)
**Patients (n = 60)**	
Age, median (IQR)	4.9 mo (10 d to 7.5 y)
Sex	
Female	31 (52)
Male	29 (48)
Medical admission	31 (52)
Cardiac diagnosis	
Single ventricle	17 (28)
Cardiomyopathy	10 (17)
LVOTO	9 (15)
Cyanotic mixing	8 (13)
Increased pulmonary blood flow	7 (12)
RVOTO	4 (7)
Other^a^	5 (8)
Congenital comorbidity (genetic or noncardiac)	16 (27)
Premature	14 (23)
In the week prior to death	
Invasive procedure(s)	25 (42)
ECMO and/or VAD	27 (45)
Open chest	16 (27)
Never or rarely aware	43 (72)
Bedbound^b^	41 (69)
Chest compressions	14 (23)
No. of invasive procedures, ECMO or VAD, open chest, unaware, immobile, or chest compressions in week prior to death	
0	5 (8)
1	12 (20)
2	8 (13)
3	13 (22)
4	12 (20)
5	9 (15)
6	1 (2)
Therapies on day of death	
Invasive ventilation	55 (92)
≥2 inotropes	46 (77)
≥2 sedatives	38 (63)
Paralytics	40 (67)
No. of invasive ventilation, ≥2 inotropes, ≥2 sedatives, or paralytics on day of death	
0	2 (3)
1	5 (8)
2	11 (18)
3	16 (27)
4	26 (43)
Medical intensity on day of death^c^	
Low	27 (45)
High	33 (55)
Mode of death	
Discontinuation of therapy	39 (65)
Limitation to therapy	10 (17)
Cardiopulmonary resuscitation	8 (13)
Comfort care only	2 (3)
Brain death	1 (2)
Resuscitation order or advance care plan	21 (35)
Pediatric palliative care involvement	32 (53)
**Staff (N = 713)**	
Discipline^d^	
Medical practitioner	208 (29)
CICU attending	71 (10)
Nurse practitioner	64 (9)
CICU fellow (cardiology, NICU, critical care trainees)	61 (9)
Cardiac surgeon (attending/senior fellow)	4 (1)
Cardiology attending	3 (<1)
Other ICU attending	5 (1)
Registered nurse	246 (35)
Allied health staff	259 (36)
Respiratory therapist	138 (19)
Child life therapist	50 (7)
Social work	32 (4)
Chaplain	18 (3)
Palliative care team	4 (1)
Nutrition	4 (1)
Interpreter	3 (<1)
Music therapist or resource specialist	4 (1)
Not recorded	6 (2)
Pediatric critical care experience, y^e^	
<2	112 (16)
2-5	186 (26)
5-10	145 (20)
10-15	95 (13)
>15	174 (24)

Abbreviations: CICU, cardiac intensive care units; ECMO, extracorporeal membrane oxygenation; ICU, intensive care unit; LVOTO, left ventricle outflow tract obstruction; NICU, neonatal intensive care unit; RVOTO, right ventricle outflow tract obstruction; VAD, ventricular assist device.

^a^ Other included, eg, cardiac transplant.

^b^ Compared with patients who were able to be maintained in a chair or be held.

^c^ Low intensity indicates no therapies, inotropes without respiratory support, and noninvasive ventilation or invasive ventilation a maximum of 1 inotrope. High intensity indicates invasive ventilation with more than 1 inotrope, having an open chest, receiving ECMO, or receiving cardiopulmonary resuscitation.

^d^ Staff involved in more than 1 patient death could respond multiple times. Nurse practitioner included 62 CICU and 2 consult NPs.

^e^ One respondent did not answer.

### PICU-QODD Survey Instrument Modified for the Cardiac Intensive Care Unit

The PICU-QODD instrument was modeled on the adult QODD questionnaire, which has been revised and studied for validated use by critical care clinicians.^18,19,23,24,25^ The PICU version better captures contextual differences in pediatric end-of-life care following modifications based on clinician and bereaved family interviews (eMethods in the Supplement).^19^ The final PICU-QODD construct comprises 20 questions (Table 2) evaluating clinician perceptions of the child’s dying process, death, and time after death with each item scored on an 11-point Likert scale (0, terrible; 10, ideal). PICU-QODD was shown to be a reliable and valid PICU clinician measure of QODD.^19^

**Table 2.** Survey Questions and Responses

Question	Median (IQR)	No.
PICU-QODD score questions^a^		
The child was free of pain	8 (7-10)	676
The child was free of other troubling symptoms	8 (5-10)	666
Clinical staff responded quickly to parents’ concern about their child’s symptoms	10 (8-10)	653
Clinical staff gave parents information about their child in a way that they could understand	10 (8-10)	664
Clinical staff prepared parents for what might happen to their child	9 (8-10)	666
Clinical staff discovered and respected parents’ wishes and decisions	10 (8-10)	681
Clinical staff created an atmosphere in which parents felt comfortable asking questions about their child	10 (9-10)	670
Clinical staff offered parents opportunities to discuss options about their child’s care with the health care team	10 (8-10)	662
There were no conflicts between parents and clinical staff about the best way to care for the child	9 (7-10)	646
Clinical staff provided parents with privacy with their child near the end of their child’s life^b^	10 (9-10)	436
Parents could easily meet their basic physical needs (accessible bathroom, showers, affordable meals, places to stay, parking, etc)	9 (8-10)	575
Clinical staff demonstrated that they cared about the child as an individual	10 (9-10)	704
Clinical staff supported the parents emotionally	10 (9-10)	689
Clinical staff provided parents with opportunities to be near their child	10 (9-10)	691
Clinical staff helped parents find ways to touch, hold, and/or connect with their child	10 (9-10)	680
Hospital clergy and chaplains were available^b^	10 (10-10)	315
Staff discovered and respected the family’s spiritual and/or religious needs	10 (9-10)	552
Staff did a good job of passing information about the child onto the next shift/rotation	10 (9-10)	635
Clinical staff helped parents create memories (such as handprints, lockets of hair, photographs) of their child^b^	10 (10-10)	380
Once the child died, his/her parents were allowed to stay with him/her for as long as they wanted^b^	10 (10-10)	306
Standardized PICU-QODD score^a^	92.5 (84.4-96.9)	637
Additional questions relating to end-of-life circumstances		
Was the mode-of-death aligned with the families wishes? No. (%)		
Yes	516 (72)	713
No	61 (9)	713
Unsure	136 (19)	713
Global rating question 1: how would you rate the quality of the patient's life during the last 7 days of his/her life?^c^	5 (2-7)	656
Global rating question 2: how would you rate the quality of the patient's moment of death?^b^^,^^c^	9 (7-10)	281

Abbreviation: PICU-QODD, pediatric intensive care unit quality of dying and death.

^a^ Responses to these questions from the PICU-QODD were used to calculate the standardized score (range, 0 to 100) obtained by summing individual scores for each question, dividing by the number of questions answered, and multiplying by 10. Responses to more than 80% of the PICU-QODD instrument questions were required for standardized score calculation. Each item is scored on an 11-point Likert scale, with 0 indicating terrible and 10 indicating ideal or near perfect.

^b^ Could respond unsure if not present or not applicable.

^c^ Rated on a Likert scale (0-10), with higher scores indicating higher quality.

For this CICU study, we made several PICU-QODD instrument modifications based on pilot data and to incorporate suggestions from prior instrument use.^19,23,26,27^ First, to minimize ceiling effect, a different Likert response label was implemented to achieve a score of 10 (ideal or near perfect rather than as good as it could be). Second, acknowledging contemporary focus on end-of-life goal-concordant care,^20,28^ we introduced an ad hoc question to ascertain knowledge of and alignment of the mode of death with the family’s wishes (yes, no, or unsure) as well as 2 global rating questions querying quality of the moment of death and quality of life in the 7 days prior (Likert scale, 0-10).^18,19^ Finally, staff had the opportunity for free text responses.

A standardized PICU-QODD score (range 0 to 100) was calculated for surveys with at least 80% responses by summing individual item scores, dividing by the number of questions answered, and multiplying by 10.^18,19^ As the PICU-QODD score was validated in the PICU population, we first attempted to validate the instrument in the pediatric CICU. We assessed performance by reporting proportions of surveys with an invalid score, a score of 0 or 100, and skewness of score distribution. Commonly used criteria suggest that less than 15% of surveys should be missing responses, less than 15% should take a value of 0 or 100, and skewness should not exceed 2.^29^ Internal consistency of individual items was assessed using Cronbach α. To evaluate construct validity, we examined Spearman correlations between the standardized score and global quality ratings; we would expect these measures to be positively correlated. We also calculated standardized scores for surveys on which respondents reported that the death did vs did not align with family wishes; we would expect that the median score should be higher for aligned deaths.

### End-of-Life Circumstances

Patient-level data collected from electronic medical records included demographic characteristics, diagnoses, palliative care involvement, and medical therapies used over the 7 days prior to death. Medical therapies included cardiopulmonary resuscitation (CPR), mechanical support (ECMO or ventricular assist device [VAD]), invasive or noninvasive ventilation (NIV), level of awareness, sedative and paralytic infusions, mobility (ambulatory, chair or bed bound), inotropes, and enteral nutrition (oral or tube). Medical intensity of end-of-life care was dichotomized as low or high. Low intensity included patients not requiring CPR, ECMO, or VAD; inotropes without respiratory support; or NIV or invasive ventilation with a maximum of 1 inotrope. High intensity was defined as requiring invasive ventilation with more than 1 inotrope, having an open chest, or receiving ECMO or CPR during the 24 hours prior to death. Mode of death was categorized as maximal resuscitation with CPR, treatment limitation (death despite ongoing cardiorespiratory support), discontinuation of life-sustaining therapy (active cessation of cardiorespiratory support), comfort care only (no interventions), and brain death.^3^

### Statistical Analysis

We summarized categorical variables using frequencies and percentages, ordinal scores using medians and IQRs, and continuous variables using means and standard deviations or medians and IQRs. The standardized PICU-QODD score was the primary outcome.^19^ Secondary outcomes included individual instrument questions evaluating pain control, symptom management, and conflict; global rating questions; and alignment. Associations between survey responses and patient and staff characteristics were evaluated using univariate regression analyses with Huber-White estimates of the model coefficient SEs to account for correlation among multiple surveys completed for the same patient. Linear regression was used for the standardized PICU-QODD score and ordinal regression for individual items. To evaluate potential confounding because of missing data and response bias, sensitivity analyses were performed calculating PICU-QODD scores using all surveys and surveys with greater than 50% completion rate, as well as bivariate models adjusting for discipline. Unit of analysis is at the decedent level and all survey data analyses account for within-patient correlation. Analyses were performed in Stata version 16 (StataCorp). All tests were 2-sided and conducted at the .05 level of significance.

## Results

### Patient Characteristics and End-of-Life Circumstances

Sixty patient deaths occurred during the study (31 [52%] female; median [IQR] age, 4.9 months [10 days to 7.5 years]) (Table 1). Thirty-three patients (55%) received high-intensity end-of-life care. In the week prior to death, patients commonly experienced CPR (14 [23%]), experienced ECMO or VAD (27 [45%]), or underwent invasive procedures (25 [42%]). The most common mode-of-death was discontinuation of life-sustaining therapy (39 [65%]).

### PICU-QODD Survey Responses

For 60 deaths, 713 surveys were completed of 994 distributed (72% response rate; median [IQR] of 11 [5-20] surveys per death). Disciplines and clinical experience of staff completing surveys and nonrespondents are shown (Table 1 and eTables 1 and 2 in the Supplement). Respondents included 246 (35%) nursing staff, 208 (29%) medical practitioners, and 259 (36%) allied health staff. Response rates were highest for nursing staff (85%) compared with medical and allied health professionals (66% and 67%, respectively) (eTable 1 in the Supplement). A broad range of critical care experience was represented (eg, 298 staff [42%] with ≤5 years). Of completed surveys, 637 (89%) had calculatable PICU-QODD standardized scores (median [IQR], 93 [84-97]).

Table 3 summarizes performance, internal consistency, and construct validity of the PICU-QODD in the CICU environment by discipline. Skew was less than −2.5, and for fellows, nurses, respiratory therapists, child life specialists, and social workers, 10% of scores were less than 80. Cronbach α showed acceptable reliability, and scores showed moderate to strong positive correlation for moment of death, fair positive correlation for the quality of 7 days prior, and higher scores were observed when death aligned with family wishes.

**Table 3.** PICU-QODD Performance, Internal Consistency, and Construct Validity in the Cardiac Intensive Care Unit by Discipline

PICU-QODD survey CICU validation	Medical practitioner (n = 208)	CICU attending (n = 71)	CICU nurse practitioner (n = 62)	Fellow (n = 61)	Nursing staff (n = 246)	Allied health (n = 259)	Respiratory therapy (n = 138)	Child life therapy (n = 50)	Social work (n = 32)
Performance and internal consistency									
Missing, No. (%)	15 (7.2)	6 (8.5)	4 (6.5)	5 (8.2)	16 (6.5)	45 (17.4)	19 (13.8)	14 (28.0)	1 (3.1)
Score of 0, No. (%)	0	0	0	0	0	0	0	0	0
Score of 100, No. (%)	21 (10.1)	8 (11.3)	6 (9.7)	4 (6.6)	20 (8.1)	16 (6.2)	6 (4.4)	0	2 (6.3)
Mean (SD)	91.9 (7.8)	92.6 (7.9)	91.8 (7.4)	90.8 (7.8)	88.3 (10.6)	88.9 (9.6)	90.2 (8.5)	86.1 (7.8)	82.0 (12.0)
Skew	−1.5	−2.3	−1.2	−1.0	−1.5	−1.1	−1.2	−1.0	−0.8
Median score	94.1	94.2	94.3	92.6	91.5	90.8	92.7	88.3	82.5
25th percentile	89.0	90.6	89.3	86.9	82.7	82.5	86.1	80.3	77.0
10th percentile	81.9	83.9	80.7	78.2	74.6	76.5	77.6	76.3	69.4
Minimum score	58.4	58.2	67.8	70.6	39.3	50.0	60.6	62.1	50.0
Cronbach α	0.87	0.90	0.32	0.72	0.90	0.92	0.89	0.91	0.95
Construct validity, coefficient (95% CI)									
Quality of life over 7 d^a^	0.32 (0.18 to 0.44)	0.40 (0.16 to 0.59)	0.15 (−0.12 to 0.40)	0.37 (0.11 to 0.58)	0.31 (0.19 to 0.42)	0.39 (0.26 to 0.50)	0.50 (0.34 to 0.62)	0.37 (0.02 to 0.63)	0.29 (0.08 to 0.59)
Quality of moment of death^a^	0.67 (0.55 to 0.75)	0.72 (0.55 to 0.83)	0.47 (0.10 to 0.72)	0.67 (0.43 to 0.82)	0.62 (0.45 to 0.74)	0.80 (0.70 to 0.87)	0.75 (0.57 to 0.85)	0.32 (0.51 to 0.83)	0.93 (0.76 to 0.98)
Alignment, median (IQR)									
Yes	94.7 (90.5 to 98.2)	95.0 (92.9 to 98.9)	95.1 (90.0 to 98.0)	93.2 (89.0 to 97.0)	92.9 (86.9 to 96.5)	92.7 (84.4 to 97.6)	93.8 (87.5 to 97.6)	88.5 (84.4 to 91.1)	83.3 (78.6 to 90.5)
No	90.3 (83.8 to 92.8)	83.8 (75.3 to 88.6)	91.7 (90.3 to 95.3)	NA	85.0 (79.4 to 93.9)	86.9 (77.0 to 91.0)	88.4 (86.0 to 94.0)	81.3 (77.0 to 90.6)	77.0 (73.2 to 82.5)

Abbreviations: CICU, cardiac intensive care units; NA, not applicable; PICU, Pediatric Intensive Care Unit; QODD, quality of dying and death.

^a^ Spearman correlation coefficients.

Quality of life in the last 7 days of life was the lowest rated item (median [IQR], 5 [2-7]; 656 total responses), with 5 the mode (121 responses [18%]) and 0 (terrible) the next most frequent response (82 responses [13%]). In contrast, the moment of death was positively perceived (median [IQR] score, 9 [7-10]; 281 responses) with 10 (ideal) the mode (111 [40%]). The lowest individual PICU-QODD item scores related to presence of pain (median [IQR], 8 [7-10]) or troubling symptoms (median [IQR] score, 8 [5-10]). Mode of death was commonly aligned with perceived families wishes (516 of 577 responses [89%]).

### Factors Associated With PICU-QODD Scores

Compared with medical practitioners, mean (SD) standardized PICU-QODD scores were 3.5 and 3.0 points lower for nursing and allied health staff, respectively (medical practitioner: 91.9 [7.8]; nursing staff: 88.3 [10.6]; allied health: 88.9 [9.6]; both *P* < .001) (Figure, A; Table 3). Compared with staff with more than 15 years’ experience, scores were 3.6 and 3.1 points lower for staff with less than 2 and 2 to 5 years of experience, respectively (<2 y: 87.7 [8.9]; 2-5 y: 88.2 [11.0]; >15 y: 91.3 [8.3]; <2 y vs >15 y: *P* < .001; 2-5 y vs >15 y: *P* = .01 (Table 3; Figure, B). When mode of death was perceived as misaligned with family wishes, scores were 7.2 points lower (mean [SD] score, aligned: 91.5 [8.3]; misaligned: 84.3 [11.4]; *P* < .001). Scores for patients with genetic or noncardiac congenital comorbidities (vs no congenital comorbidities) were lower (mean [SD], with comorbidities: 87.0 [11.8]; no comorbidities: 90.5 [8.5]; *P* = .046), as were those with surgical (vs medical) admissions (mean [SD] score, surgical: 88.2 [9.9]; medical: 90.9 [9.1]; *P* = .04). Compared with discontinuation of life-sustaining therapy (mean [SD] score, 90.8 [8.8]), scores were 5.0 points lower when death followed treatment limitation (mean [SD] score, 85.8 [10.3]; *P* = .008). Involvement of palliative care, high intensity of medical care, and ECMO or invasive procedures prior to death were not significantly associated with PICU-QODD scores (Table 4).

**Figure.**
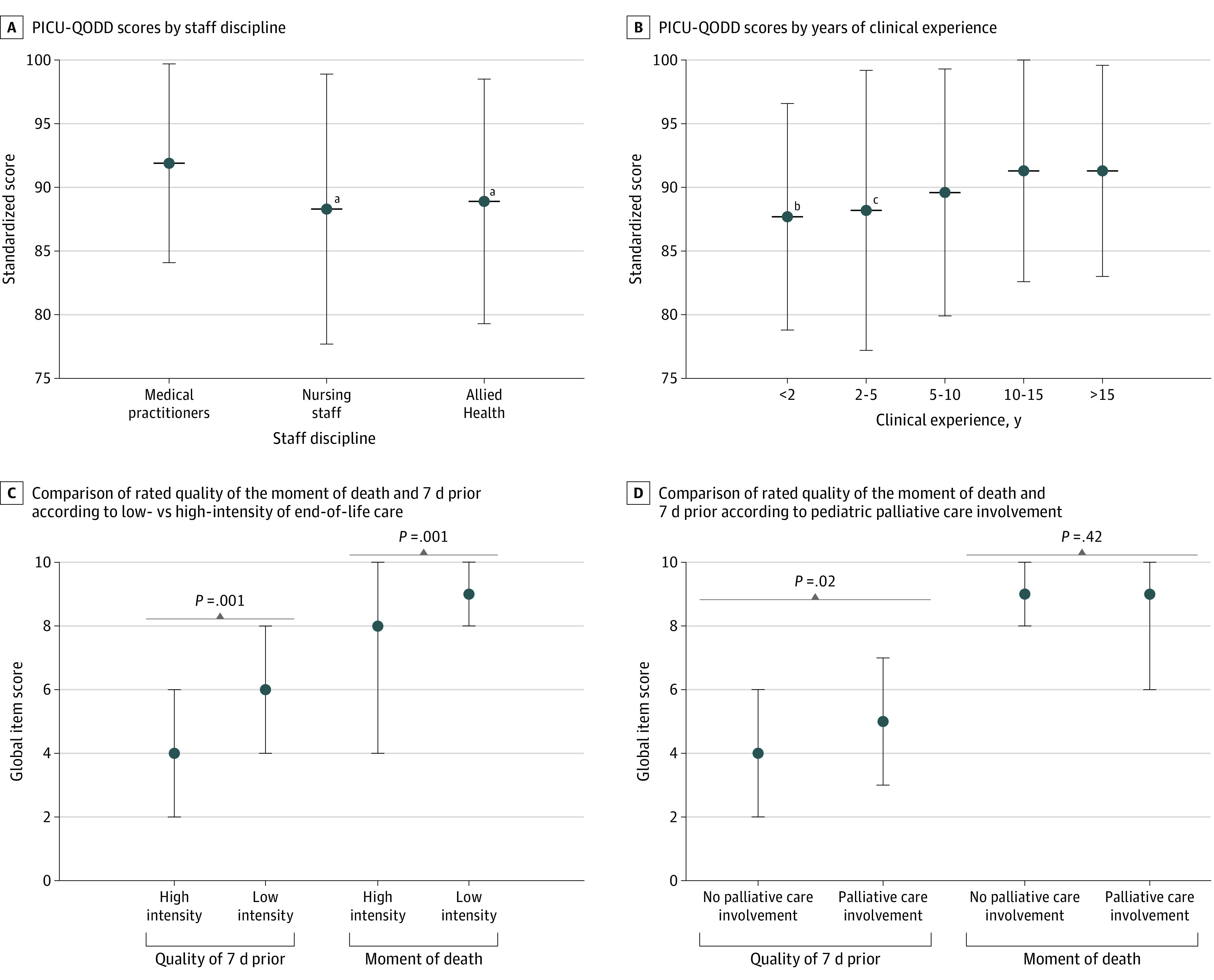
Comparison of Standardized Pediatric Intensive Care Unit Quality of Death and Dying (PICU-QODD) Scores by Staff Discipline, Years of Pediatric Critical Care Experience, and the Moment of Death vs 7 Days According to Intensity of End-of-Life Care and Pediatric Palliative Care Involvement Dot indicates median and whiskers, IQRs. ^a^*P* < .001 relative to medical practitioners. ^b^*P* < .001 relative to those with more than 15 years clinical experience. ^c^*P* = .01 relative to those with more than 15 years clinical experience.

**Table 4.** Mean Standardized PICU-QODD Score and Univariate Linear Regression Models^a^

Variable	Responses, No. (%) (N = 637)	QODD score, mean (SD)	Coefficient (95% CI)
Admission category			
Medical	329 (52)	90.9 (9.1)	[Reference]
Surgical	308 (48)	88.2 (9.9)	−2.6 (−5.2 to −0.1)
Congenital comorbidity			
Absent	474 (74)	90.5 (8.5)	[Reference]
Genetic or noncardiac anomaly	163 (26)	87.0 (11.8)	−3.5 (−7.0 to −0.1)
Medical intensity at end-of-life^b^			
Low	272 (43)	90.5 (8.6)	[Reference]
High	365 (57)	89.0 (10.2)	−1.5 (−4.1 to 1.1)
Mode of death			
Withdrawal of therapy	428 (67)	90.8 (8.8)	[Reference]
Limitation to therapy	103 (16)	85.8 (10.3)	−5.0 (−8.7 to −1.3)
CPR	78 (12)	86.7 (11.5)	−4.0 (−8.9 to 0.8)
Comfort care only	15 (2)	93.1 (7.2)	2.3 (−2.9 to 7.5)
Brain death	13 (2)	94.8 (5.6)	4.1 (2.8 to 5.4)
Palliative care involvement			
No palliative care	290 (46)	89.5 (10.2)	[Reference]
Palliative care involved	347 (54)	89.7 (9.2)	0.1 (−2.6 to 2.8)
Death perceived to be aligned with family’s wishes			
Yes	479 (75)	91.5 (8.3)	7.2 (4.4 to 10.0)
No	54 (8)	84.3 (11.4)	[Reference]
NA	104 (16)	83.8 (10.7)	−0.5 (−3.7 to 2.7)
Discipline			
Medical	193 (30)	91.9 (7.8)	[Reference]
Nursing	230 (36)	88.3 (10.6)	−3.5 (−5.3 to −1.8)
Allied	214 (34)	88.9 (9.6)	−3.0 (−4.5 to −1.5)
Pediatric critical care experience, y			
<2	104 (16)	87.7 (8.9)	−3.6 (−5.6 to −1.6)
2-5	161 (25)	88.2 (11.0)	−3.1 (−5.4 to −0.7)
5-10	126 (20)	89.6 (9.7)	−1.7 (−3.9 to 0.5)
10-15	87 (14)	91.3 (8.9)	0.0 (−2.0 to 2.0)
>15	158 (25)	91.3 (8.3)	[Reference]

Abbreviations: CPR, cardiopulmonary resuscitation; ECMO, extracorporeal membrane oxygenation; NA, not applicable; PICU, pediatric intensive care unit; QODD, quality of dying and death.

^a^ Accounting for the correlation of multiple surveys within each patient.

^b^ Low intensity indicates no therapies, inotropes without respiratory support, and noninvasive ventilation or invasive ventilation with a maximum of 1 inotrope. High intensity indicates invasive ventilation with more than 1 inotrope, having an open chest, receiving ECMO, or receiving CPR.

### Subanalyses of Individual Question

High medical intensity was associated with lower median [IQR] rated quality of the 7 days prior to death and at the moment of death compared with low medical intensity (7 days prior: 4 [2-6] vs 6 [4-8]; *P* = .001; moment of death: 8 [4-10] vs low intensity 9 [8-10] *P* = .001) (Figure, C). Median (IQR) quality of life for the 7 days prior to death was rated lower for those without vs with palliative care involvement (4 [2-6] vs 5 [3-7]; *P* = .02) (Figure, D). Nursing staff recorded lower median (IQR) quality of life scores during the 7 days prior to death compared with medical staff (4 [2-6] vs 5 [3-7]; *P* = .002), while there was no statistical difference between scores from medical and allied health staff (*P* = .42) (eFigure in the Supplement). The moment of death was rated similarly highly by all disciplines (eFigure in the Supplement). Responses to individual PICU-QODD instrument questions relating to pain, troubling symptoms, and conflict had similar differences according to staff discipline and critical care experience as the standardized score.

### Sensitivity Analyses

Similar associations and coefficients were observed in sensitivity analyses using varying survey completion criteria for standardized PICU-QODD calculation and bivariate models adjusting for discipline (eTables 3 and 4 in the Supplement).

## Discussion

In this cross-sectional survey study to assess CICU interdisciplinary team’s insights into what constitutes high-quality end-of-life care for dying children with heart disease, the PICU-QODD instrument shows promise as a reliable and valid measure. We observed overall positive perceptions of QODD, particularly the moment of death, yet the 7 days prior were negatively perceived. Significantly lower scores were reported by nursing or allied health staff compared with medical practitioners, by less experienced staff, for patients with cardiac-surgical admissions, for patients with comorbidities, and for deaths following treatment limitation and/or misaligned with the family’s wishes. We observed lower ratings of the quality of the moment of death and 7 days prior in patients who received high-intensity medical care at end of life. Palliative care involvement was associated with higher perceived quality of life in the 7 days prior to death.

While defining high-quality end-of-life care is a key research priority, no metrics focus on the unique needs of the CICU population.^2,11,15,20,28,30^ The QODD instrument offers a means to evaluate clinician and family perceptions and has been studied in more than 4000 adult deaths across multiple settings, populations, and countries, gaining prominence as an outcome measure in end-of-life care.^23,24,25,26,31,32^ The PICU-QODD version aimed to fill this gap for critically ill children.^19,30^ Despite differences in pediatric CICUs and PICUs, we demonstrated utility of the PICU-QODD instrument as a clinician measure of QODD in the CICU environment. To our knowledge, this is the largest study evaluating clinician perceptions of QODD in critically ill children. Findings align with observations in PICUs,^19^ suggesting a potential role for this instrument as a universal clinician measure of QODD in pediatric critical illness. This means that similar to the adult tool,^18,23,24,25,26,27,33,34,35^ comparing QODD in children across settings may be feasible for future research to guide quality of end-of-life improvement strategies.^26^ Shortcomings identified in our study and others include ceiling effects, inability to respond to certain items relating to presence, and lack of score increment following interventions.^26,36,37,38^ While no well-defined clinically significant difference for QODD exists, standardized scores lower than 85 in the lowest 25th percentile across disciplines have been consistent findings in pediatrics, and the observed 7-point increment in scores with family alignment in our study are equivalent to adult studies, both of which could represent quality improvement targets.^18,19,26,37^ High scores likely represent quality end-of-life care; however, modifications to the response labels (ie, additional midrange terms, such as could be improved, and using perfect for a 10-point score) may augment the ability to determine clinically meaningful differences. Given known challenges obtaining data from bereaved family members, there are clear benefits using instruments evaluating clinician perspectives as a proxy.^13,19,21^ As CICU staff have likely experienced multiple patient deaths (relative to families), their comparative perspectives are highly valuable. Availability of a validated, reliable outcome measure will help drive efforts to improve quality of end-of-life care. Furthermore, staff well-being has been named a health care crisis.^39,40^ End-of-life care is a frequent driver of moral injury, demanding consideration of team perceptions.^6^ The impressive response rate indicates that CICU team members are eager and willing to share their insights. Further research using the PICU-QODD instrument is needed to confirm utility as a core pediatric end-of-life outcome measure, explore correlation with family bereavement outcomes, and the impact on staff well-being.

The quality of dying and death construct focuses on the final stage of illness and death experience.^18,19^ Divergence in clinician’s perceptions of QODD overall and moment of death vs the 7 days prior warrants further exploration. These findings are consistent with bereaved parent survey results, in which 50% described their child’s overall quality of life in the last month as poor or fair, while 70% of parents agreed their child experienced a good death.^13,21^ This implies both staff and parents and guardians perceive burdens of intensive interventions yet tolerate them when death is the alternative.^5^ These findings raise the question as to whether this high-risk, high-reward strategy may be negatively reflected upon after death for both staff and parents and guardians.^2,3,15,20^ Perceptions of higher quality of death following discontinuation of life-sustaining therapy compared with limitations of therapy may be attributable to greater certainty and control over the timing when death is imminent. In adult ICUs, data supports early palliative care integration and less invasive interventions result in better end-of-life experience for staff.^41,42^ Palliative care involvement in CICUs results in less intense therapies at end-of-life.^3,15,20^ Both these factors were associated with positive perceptions of quality of life in the 7 days prior to death in our study, and QODD was perceived highly when staff felt mode of death was aligned with the family’s wishes. This reinforces prioritizing goal-concordant care and suggests that interventions focused integrating palliative care principles concurrent with disease-directed therapy and augmenting team communication surrounding family’s goals of care may improve staff perceptions of the dying experience.^20,28,43^

Despite high overall scores, our findings highlight a disconnect between perceptions of CICU QODD according to discipline and clinical experience. These observations are reflected in other critical care settings, although limited data exist for allied health professionals.^18,19,23,41,44^ Variable perception across interdisciplinary staff may relate to different lengths of time with a given patient, diversity of experiences, and events witnessed during ICU admissions.^23^ In our CICU, nurses along with a respiratory therapist (responsible for ventilatory and ECMO care), manage moment-to-moment care and directly perform invasive interventions. Thus, it is understandable that staff spending the greatest amount of time at the bedside more negatively perceive QODD. Our findings of alignment with family wishes strongly influencing perceptions further substantiate how family fears surrounding the death of their loved one and unrealistic expectations significantly contribute to staff distress.^45^ Nurses with less clinical experience also tend to perceive high levels of stress and emotional responses to dying and death.^46,47,48^ This may relate to lack of previous dying or death experiences or inadequate preparatory training.^19,46^ Traditionally, nursing education programs have focused on health promotion with less preparation in coping with and managing end of life.^46,47,48^ In response, international nursing organizations have emphasized importance of robust palliative care instruction in curricula.^46^ Simulation training may better prepare staff with effective communication and clinical skills.^49^ Finally, nursing and allied health staff may experience frustration about their limited role and participation in end-of-life decision-making.^23,41,44^ Interdisciplinary team support and institutional practices including joint approaches to symptom control, routine team meetings, ethical competency training, and robust discussions sharing perspectives may overcome these challenges.^41,43,50,51,52^

### Limitations

This study has limitations. It was conducted in a single-center quaternary US institution known for innovation, limiting generalizability. While the overall response rate for end-of-life survey methods was remarkable and sensitivity analyses suggest our findings would not change substantially if more surveys had been completed, results could be influenced by responder and recall bias. Also, because we were unable to account for correlation among the responses given by the same health care professional, SEs of regression coefficients might be underestimated. Although end-of-life care is a recognized driver of moral distress and burnout, with most ethical issues and conflict in critical care settings arising in this context,^6,51^ we did not co-assess these scores owing to time constraints to increase survey completion. Additionally, we intended to evaluate staff perceptions and acknowledge this study does not reflect parent and guardian perceptions or prospective symptom assessment.

## Conclusions

In this cross-sectional survey study of CICU staff, the PICU-QODD instrument was found to be a reliable and valid clinician measure of QODD. CICU interdisciplinary staff positively perceived QODD and quality of the moment of death yet had a negative perception of the 7 days prior. Significantly lower scores were reported by nursing or allied health professionals, staff with less clinical experience, and patients who received high-intensity care at end-of-life. More data are needed to establish PICU-QODD as a core end-of-life outcome measure. Our findings offer an opportunity to guide strategies to meaningfully improve staff well-being and patient and family end-of-life experiences.

## Notes

Supplement. **eMethods. **Expanded Methods **eTable 1. **Staff Discipline Data With Comparison of Respondents and Nonrespondents **eTable 2. **Clinical Experience Data With Comparison of Respondents According to the Meeting Required Numbers of Responses for Calculation of Standardized QODD Scores **eTable 3. **Comparison of QODD Scores Including All Survey Respondents and According to the Meeting Required Numbers of Responses for Calculation of Standardized QODD Scores **eTable 4. **Bivariate Linear Regression Models for the Association Between Standardized PICU-QODD Score and End-of-Life Circumstances and Staff Clinical Experience, Adjusting for Professional Role **eFigure. **Box and Whisker Plot Demonstrating Comparison of Rated Quality of the Moment of Death and 7 Days Prior Using a Likert Scale (0, Terrible; 10, Ideal) Overall and by Staff Discipline
