# Supplementary material for: Assessment of an Instrument to Measure Interdisciplinary Staff Perceptions of Quality of Dying and Death in a Pediatric Cardiac Intensive Care Unit
Source: JAMA Netw Open. 2022 May 6;5(5):e2210762. doi: 10.1001/jamanetworkopen.2022.10762 (PMC9077481; doi:10.1001/jamanetworkopen.2022.10762)
Supplement: Supplement. — eMethods. Expanded Methods eTable 1. Staff Discipline Data With Comparison of Respondents and Nonrespondents eTable 2. Clinical Experience Data With Comparison of Respondents According to the Meeting Required Numbers of Responses for Calculation of Standardized QODD Scores eTable 3. Comparison of QODD Scores Including All Survey Respondents and According to the Meeting Required Numbers of Responses for Calculation of Standardized QODD Scores eTable 4. Bivariate Linear Regression Models for the Association Between Standardized PICU-QODD Score and End-of-Life Circumstances and Staff Clinical Experience, Adjusting for Professional Role eFigure. Box and Whisker Plot Demonstrating Comparison of Rated Quality of the Moment of Death and 7 Days Prior Using a Likert Scale (0, Terrible; 10, Ideal) Overall and by Staff Discipline [file jamanetwopen-e2210762-s001.pdf]

Supplemental Online Content

Bailey V, Beke DM, Snaman JM, et al. Assessment of instrument to measure interdisciplinary staff perceptions of quality of dying and death in a pediatric cardiac intensive care unit. *JAMA Netw Open*. 2022;5(5):e2210762. doi:10.1001/jamanetworkopen.2022.10762

eMethods. Expanded Methods

eTable 1. Staff Discipline Data With Comparison of Respondents and Nonrespondents

eTable 2. Clinical Experience Data With Comparison of Respondents According to the Meeting Required Numbers of Responses for Calculation of Standardized QODD Scores

eTable 3. Comparison of QODD Scores Including All Survey Respondents and According to the Meeting Required Numbers of Responses for Calculation of Standardized QODD Scores

eTable 4. Bivariate Linear Regression Models for the Association Between Standardized PICU-QODD Score and End-of-life Circumstances and Staff Clinical Experience, Adjusting for Professional Role

eFigure. Box and Whisker Plot Demonstrating Comparison of Rated Quality of the Moment of Death and 7 Days Prior Using a Likert Scale (0, terrible; 10, ideal) Overall and by Staff Discipline

This supplemental material has been provided by the authors to give readers additional information about their work.

© 2022 Bailey V et al. *JAMA Network Open.*

eMethods. Expanded Methods:

Summary of QODD Construct Development:

In the 1990s, Curtis et al developed a 31-item measure of the quality of dying and death using focus groups and interviews with patients, family members, and clinicians; and a literature review[2]. The original Quality of Dying and Death (QODD) questionnaire had two versions designed for completion after death by family members or clinicians[3,4]. The family QODD has two parts to each question. Part A evaluates the frequency with which an experience occurred, and Part B of each question asks the family to rate the quality. The clinician version of the QODD instrument contained rating items assessing the quality of each experience on a 0 to 10 scale, with a score of 0 designating a “terrible experience,” and 10 an “almost perfect experience.” The tool was adapted for use in the ICU deleting 10 items that had limited relevance to the critical care setting, low completion rates, and lack of concordance with bereaved family members[4,5]. The adult version of the QODD instrument and ICU refinements have been validated and used extensively to measure perceptions of critical care healthcare workers in numerous countries[4–10].

Sellers et al sought to create a similar instrument to measure quality of dying and death for critically ill children and the format and development of the PICU-QODD was modeled after the adult instrument[1]. As the pediatric context is substantially different from adults, the definition adopted for the PICU-QODD emphasizes the hopes and priorities of the family rather than the preferences of the patient. Additionally, most children die in the hospital setting, frequently in a critical care environment. Consequently, focus groups with clinicians who have cared for children who died and qualitative interviews with parents of children who died in a PICU were conducted to incorporate their views in the development of the measure. Of 43 items in the pilot version, following standard item analyses to assess the performance of individual questions and content revision, the final result was a 20-item instrument referred to as PICU-QODD. For psychometric purposes of construct validity, the clinician survey instrument in both constructs for adults and children included two additional single-item questions related to quality of life in the seven days prior and at the moment of death. The quality of care at the end of life, the quality of the dying and death moment, were significantly and positively correlated with the pediatric and adult versions of the standardized QODD score. In the pediatric validation very few parent/family surveys were completed so parent and clinician ratings were unable to be compared, and family completion of the survey was not validated.

The Likert scale differs among the instruments for rating the score of 10. The PICU-QODD originally used ‘as good as it could be, under the circumstances’’, initially intended to be sensitive to the parents’ grief (as both parent and clinician instruments used the same anchors) but considered this to have produced overly positive ratings. We similarly observed this in our pilot feasibility survey of 128 practitioners and 16 deaths (Pediatric Cardiac Intensive Care Society (PCICS) meeting in 2019 (Bailey V, Beke D, Snaman J, Smith-Parrish M, Thornton J, Thiagarajan R, Moynihan K. Perceptions of quality of death and dying in the Cardiac ICU. PCICS, London, November 2019). As such the authors suggested that in the final version of the PICU-QODD, the label for the score of 10 could be changed to make explicit that the comparison is an ideal experience. The adult version used ‘‘a near perfect experience’[2,3]. So, in this current study ‘ideal or near perfect’ was used..

Finally, based on pilot data response rates and feedback, two measures were instituted to increase participation, survey completion and ensure anonymity; only limited demographics were collected (years of experience both in pediatric critical care and years of post-graduate/post-bachelor/post-associate degree experience as well as discipline) and no staff level linkage was performed.

© 2022 Bailey V et al. *JAMA Network Open.*

eTable 1. Staff Discipline Data With Comparison of Respondents and Nonrespondents

Professional role	Surveys Distributed N= 994	Completed Surveys and Response Rate N= 713 (% response rate)[a]	80% of Survey Completed and Response Rate* N=637 (% response rate)[a, b]
Medical provider	315	208 (66%)	193 (61%)
CICU Attending	100	71 (71%)	65 (65%)
Nurse practitioner[c]	78	64 (82%)	60 (77%)
CICU fellow[c]	101	61 (60%)	56 (56%)
Cardiac surgeon (attending/senior fellow)	10	4 (40%)	4 (40%)
Cardiology attending	14	3 (21%)	3 (21%)
Other ICU attending	12	5 (42%)	5 (42%)
Registered Nurse	290	246 (85%)	230 (79%)
Allied Health Staff	389	259 (67%)	214 (55%)
Respiratory therapist	199	138 (69%)	119 (60%)
Child life therapist	62	50 (81%)	36 (58%)
Social Work	55	32 (58%)	31 (56%)
Chaplain	40	18 (45%)	11 (28%)
Palliative Care Team	20	4 (20%)	4 (20%)
Nutrition	5	4 (80%)	4 (80%)
Interpreter	4	3 (75%)	2 (50%)
Music Therapist/Resource Specialist	4	4 (100%)	2 (50%)
Not recorded	-	6 -	5 (-)

CICU Cardiac Intensive Care Unit, NICU Neonatal intensive Care Unit, Other ICU attending (NICU/Medical/Surgical ICU). In total the CICU comprises 175 nursing staff, 16 Attendings, 14 Nurse Practitioners and 24 Fellows.

[a] Mean response rate overall (completed verses distributed) was 72% and mean response rate limited to those with standardized scores was 64%.

[b] A prior defined criteria for calculation of the standardized PICU-QODD score was completion of >80% of the items that comprise the instrument.

[c] Fellows include cardiology, NICU, critical care trainees and NPs include consult and CICU NPs.

© 2022 Bailey V et al. *JAMA Network Open.*

eTable 2. Clinical Experience Data With Comparison of Respondents According to the Meeting Required Numbers of Responses for Calculation of Standardized QODD Scores

Pediatric critical care experience (years)	Completed Surveys (n, %), N= 713	80% of Survey Completed (n, %), N=637
<2	112 (16)	104 (16)
2-5	186 (26)	161 (25)
5-10	145 (20)	126 (20)
10-15	95 (13)	87 (14)
>15	174 (24)	158 (25)

© 2022 Bailey V et al. *JAMA Network Open.*

eTable 3. Comparison of QODD Scores Including All Survey Respondents and According to the Meeting Required Numbers of Responses for Calculation of Standardized QODD Scores

**Responses**	**Completed Surveys All Respondents**	**80% of Survey Completed**	**50% of Survey Completed**	**<80% of Survey Completed**
QODD Score<br>Median, (IQR)<br>Mean, (SD)	N= 713<br>92 (84, 97)<br>89.4 (9.8)	N=637<br>93 (84, 97)<br>89.6 (9.6)	N=688<br>92 (84,97)<br>89.5 (9.5)	N=76<br>89 (82, 95)<br>87.4 (11.3)
Quality of life (last 7 days) Median, (IQR)	N=656<br>5 (2, 7)	N=599<br>5 (2, 7)	N=643<br>5 (2, 7)	N=57<br>5 (2, 7)

© 2022 Bailey V et al. *JAMA Network Open.*

eTable 4. Bivariate Linear Regression Models for the Association Between Standardized PICU-QODD Score and End-of-life Circumstances and Staff Clinical Experience, Adjusting for Professional Role

**ETable 4 Mean Standardized PICU-QODD Score and Bivariate Linear Regression Models[a]**. N=637		
Variable	N (%)	Coefficient (95% CI)
Admission Category		
Medical	329 (52)	Ref
Surgical	308 (48)	**-2.6 (-5.1, -0.02)**
Congenital comorbidity		
Absent	474 (74)	Ref
Genetic/non-cardiac anomaly	163 (26)	**-3.6 (-7.0, -0.1)**
Medical Intensity at EOL [b]		
Low	272 (43)	Ref
High	365 (57)	-1.5 (-4.1, 1.1)
Mode of death		
Withdrawal of therapy	428 (67)	Ref
Limitation to therapy	103 (16)	**-5.1 (-8.7, -1.5)**
Cardiopulmonary resuscitation (CPR)	78 (12)	-4.2 (-9.0, 0.6)
Comfort care only	15 (2)	2.8 (-2.0, 7.5)
Brain death	13 (2)	**4.5 (3.1, 5.9)**
Subspeciality Palliative Care involvement		
No Palliative Care	290 (46)	Ref
Palliative Care	347 (54)	0.1 (-2.5, 2.8)
Death aligned with family's wishes		
Yes	479 (75)	**6.7 (3.9, 9.5)**
No	54 (8)	Ref
N/a	104 (16)	-0.7 (-3.9, 2.6)
Pediatric critical care experience (years)		
<2	104 (16)	**-3.6 (-5.6, -1.6)**
2-5	161 (25)	**-3.2 (-5.5, -1.0)**
5-10	126 (20)	-1.7 (-3.8, 0.4)
10-15	87 (14)	-0.2 (-2.2, 1.8)
>15	158 (25)	Ref

[a] Adjusting for Professional Role and accounting for the correlation of multiple surveys within each patient

eFigure. Box and Whisker Plot Demonstrating Comparison of Rated Quality of the Moment of Death and 7 Days Prior Using a Likert Scale (0, terrible; 10, ideal) Overall and by Staff Discipline

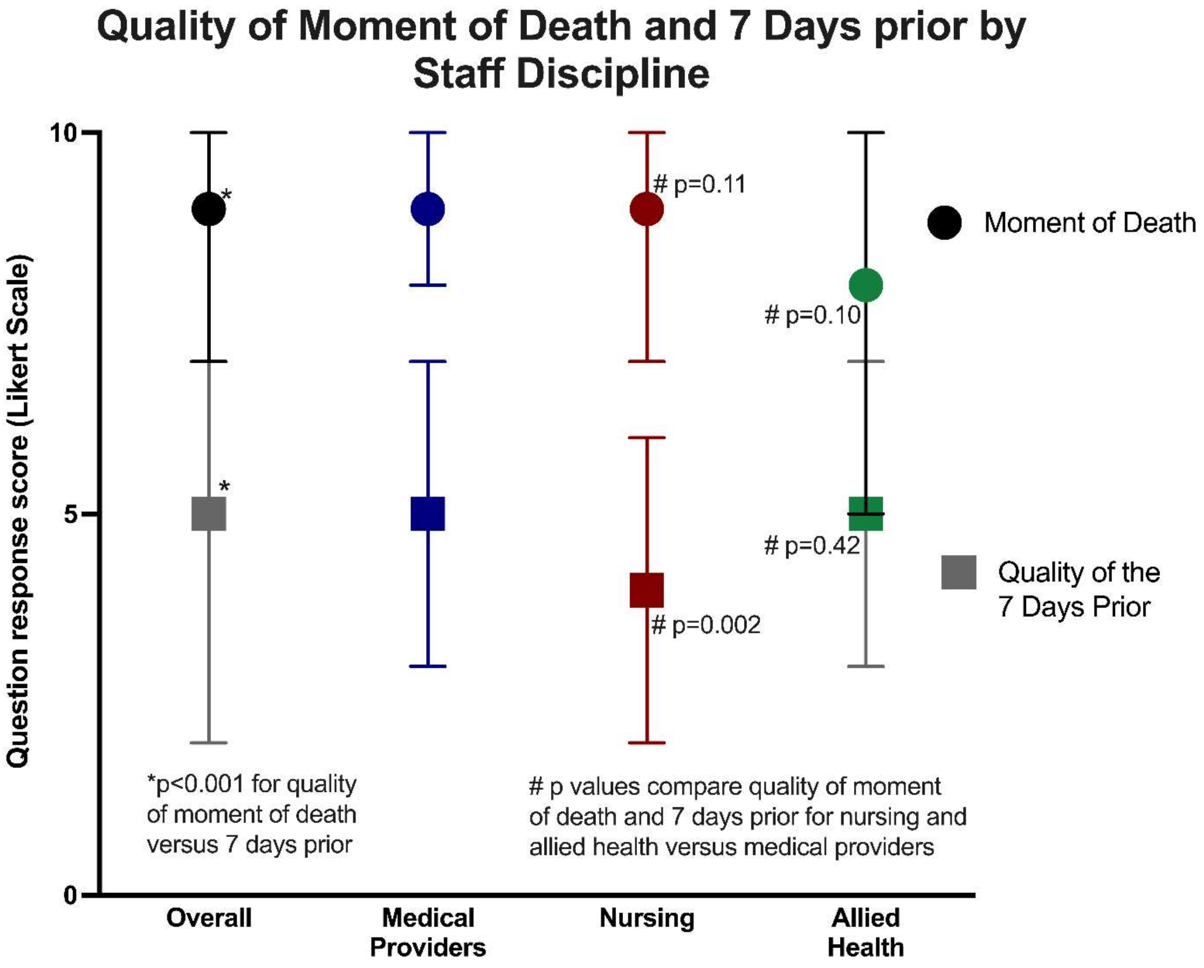

© 2022 Bailey V et al. *JAMA Network Open.*
